# Corneal epithelial permeability to fluorescein in humans by a multi-drop method

**DOI:** 10.1371/journal.pone.0198831

**Published:** 2018-06-19

**Authors:** Sangly P. Srinivas, Arushi Goyal, Deepti P. Talele, Sanjay Mahadik, Rachapalle Reddi Sudhir, P. Pavani Murthy, Sudhir Ranganath, Uday B. Kompella, Prema Padmanabhan

**Affiliations:** 1 School of Optometry, Indiana University, Bloomington, Indiana, United States of America; 2 Department of Cornea and Refractive Surgery, Sankara Nethralaya, Chennai, India; 3 Department of Chemical Engineering, Siddaganga Institute of Technology, Tumkur, India; 4 Pharmaceutical Sciences, University of Colorado Anschutz Medical Campus, Aurora, Colorado, United States of America; Save Sight Institute, AUSTRALIA

## Abstract

**Purpose:**

The permeability of the corneal epithelium to fluorescein P_dc_ is an indicator of the health of the ocular surface. It can be measured in a clinical setting by determining the accumulation of fluorescein in the stroma following administration of the dye on the ocular surface. Here we demonstrate a new multi-drop method for the measurement of P_dc_ by a spot fluorometer.

**Methods:**

Twenty-nine healthy participants were recruited for this study. First, a probe-drop of fluorescein (0.35%, 2 μL) was instilled on the conjunctiva. The clearance of the dye from the tears was immediately measured using the fluorometer. Following this, two loading drops (2%; 6 μL each) were administered 10 min apart. Fifteen minutes later, the ocular surface was washed and fluorescence from the stroma F_s_ was measured. Permeability was calculated using P_dc_ = (Q x F_s_)/ (2 x AUC), where Q is the stromal thickness and AUC is the area under the fluorescence *vs*. time curve for the loading drops.

**Results:**

After the probe drop, the tear fluorescence followed an exponential decay (elimination rate constant; k_d_ = 0.41 ± 0.28 per min; 49 eyes of 29 subjects), but the increase in F_s_ was negligible. However, after the loading drops, the measured F_s_ was ~ 20-fold higher than the autofluorescence and could be recorded at a high signal to noise ratio (SNR > 40). The intra-subject variability of k_d_ was insignificant. Since fluorescein undergoes concentration quenching at > 0.5%, the value of AUC for the loading drops was estimated by scaling the AUC of the probe drop. The calculated P_dc_ was 0.54 ± 0.54 nm/sec (n = 49). A Monte Carlo simulation of the model for the multi-drop protocol confirmed the robustness of the estimated P_dc_.

**Conclusions:**

The new multi-drop method can be used in place of the single-drop approach. It can overcome a lack of sensitivity in fluorometers of high axial resolution. The P_dc_ estimated by the multi-drop method is ~ 11-fold higher than previously reported but closer to the value reported for other drugs with equivalent octanol/water partition coefficient.

## Introduction

The corneal epithelium is the outermost layer of the cornea. It is organized, in humans, as a stratified tissue of 5–6 layers of non-keratinized cells [1]. The epithelium forms a barrier that regulates the entry of most pathogenic agents and noxious stimuli. It also restricts the entry of electrolytes and water from the tears and determines the bioavailability of hydrophilic topical drugs in the anterior chamber [2–8]. In many ocular surface disorders, including dry eye disease, the barrier integrity of the epithelium is significantly affected by pro-inflammatory cytokines [9–12]. The disruption/recovery of the epithelial barrier is also of interest in corneal wound healing [13–15], refractive surgery [16–19], limbal stem cell deficiency [20], diabetes [21–24], and in the response to chronic administration of topical drugs with preservatives (e.g., benzalkonium chloride) [25–27]. Overall, the barrier integrity of the corneal epithelium is an essential indicator of the health of the ocular surface and significantly affects the pharmacokinetics of topical drugs to the eye.

In humans, the barrier integrity of the corneal epithelium is usually expressed as permeability to fluorescein (MW: 375 Da) [27–38], which is a non-toxic fluorescent dye approved for routine ophthalmic diagnostic applications. Its octanol/water partition coefficient is very low (~ 0.04 at physiological pH) [39–41], and hence it is a hydrophilic dye capable of penetrating the corneal epithelium mainly via paracellular pathways [38, 41–43]. Thus, fluorescein is a suitable tracer for objective quantification of the barrier integrity of not only the corneal epithelium but also of other ocular epithelia [41, 44] and the vascular endothelium [45]. It must be noted, however, that our assumption that fluorescein transport is mainly via paracellular pathway can be questioned by several recent observations [46–49].

A typical protocol for the measurement of the permeability of the corneal epithelium to fluorescein (P_dc_) in humans involves topical administration of a single drop of the dye on the ocular surface, followed by measurements of its clearance from tears and its accumulation in the corneal stroma by ocular fluorometry [36, 50–54]. This method, which forms the single-drop technique, has led to P_dc_ estimates in the range of 0.04 to 0.75 nm/sec with many studies claiming a mean value around 0.05 nm/sec (i.e., 5x10^-9^ cm/sec) in healthy subjects [30–32, 34, 35, 37, 54, 55] (Table 1). An alternative to the single-drop method is the bath technique [56]. In this method, the gradient for fluorescein across the epithelium for accumulation in the stroma is held constant. This is achieved by exposing the cornea to a bath of fluorescein at a steady concentration for a finite period (~30 min) [56]. The P_dc_ estimates obtained with the bath technique [56] and the single-drop method are comparable. However, the P_dc_ estimates are small when compared to many drugs of similar molecular weight and lipophilicity (Table 2) [57–59]. Although many observations cited in Table 2 are in animal models, the extremely small value of P_dc_ remains an enigmatic observation. It is also possible that species differences could be present in the reported P_dc_ values. Similarly, the adverse effects of preservatives in breaking down the tight junctions, and thereby leading to higher values of P_dc_, also cannot be discounted.

**Table 1:** Reported values of corneal epithelial permeability to fluorescein *in vivo* in humans.

Method	Study Details	Permeability (nm/sec)	Reference
Single drop method in humans	Healthy eyes	~ 0.050	McNamara et al. [37]
Single drop method in humans	Healthy eyes Found maximum concentration at which reliable values for permeability is 0.75% fluorescein	Mean ± SE (min to max) 0.24 ± 0.04 (0.05–0.65)	Joshi et al., [29]
Single drop method in humans	Healthy eyes (After 1 hour of hypoxia).	0.067 (0.082) (median values)	McNamara et al., [35]
Bath technique	Healthy eyes	0.038 ± 0.017 (Mean ± SD)	de Kruijf et al., [56]
Single drop method in humans	Healthy eyes	0.1868	Nelson [36]
	Keratoconjunctivitis sicca patients	0.7385	
Bath technique in the rabbit *in vivo* for 5 min	Healthy eyes	0.0455	McCarey and Reaves [55]
	Tear substitutes with preservatives	0.512–0.542	McCarey and Reaves [27]

**Table 2:** Permeability of cornea to representative drugs/solutes* *vis-à-vis* their octanol-water partition coefficient (log PC)/distribution coefficient (log D) (extracted from Table 1 of Prausnitz and Noonan [58]). log(PC) of fluorescein = -1.4 [39, 40].

Drug/solute	log (PC)/log D	P_dc_(cm/sec)
Acetazolamide	-0.26/-1.31	5.1 x10^-7^
Benzolamide	0.32/-1.68	3.3x10^-7^
Bromacetazolamide	-0.02/-2.02	3.6x10^-7^
Glycerol	-2.19/-2.19	4.5x10^-6^
Mannitol	-4.67/-4.67	2.4x10^-6^
Phenylephrine	-0.72/-2.72	9.4x10^-7^
Pilocarpine	0.74/0.46	1.7x10^-5^
Timolol	1.61/-0.39	1.2x10^-5^
Tobramycin	-7.32/NA	5.2x10^-7^

* Permeability obtained from rabbit studies [58].

Of the many specifications of ocular fluorometers [7, 12, 44, 54, 60], two critical parameters include depth resolution (i.e., axial resolution) and fluorescence sensitivity. These parameters are interdependent in that attempts to obtain a higher axial resolution (e.g., by reducing excitation/emission slit widths) reduces the fluorescence sensitivity of the instrument. In other words, an increase in axial resolution results in decreased signal-to-noise ratio (SNR) of the fluorescence measurements. Although high concentrations of the dye can be employed to improve SNR, the approach would be counterproductive. At high concentrations, the dye does not get uniformly excited across the measurement depth. Since the excitation light gets absorbed by the dye, its intensity at greater depths of measurement would be diminished as per Beer-Lambert’s Law. Accordingly, the measured fluorescence from the deeper layers would be smaller than the same concentration of the dye in the upper layers. Similarly, absorption of the emission in the deeper layers also results in a further reduction in the measured fluorescence. These phenomena, which are collectively known as inner filter effects (IFEs), or static/concentration quenching, results in diminished observed fluorescence with increasing concentration of the dye [61]. Thus, concentration quenching can contribute to the nonlinearity between measured fluorescence and concentration at high levels of the dye. Therefore, the concentration of fluorescein in the administered drops has to be limited, and it is usually less than 0.5% [29, 61–63].

When we attempted to employ the single-drop protocol with our custom-made spot fluorometer, we encountered two problems: (1) the calculation method developed previously was not found to be applicable for our instrument (as there is no axial scanning involved with spot fluorometry), and (2) the accumulation of fluorescein in the stroma after one drop could not be measured with high signal-to-noise ratio (SNR). To overcome these problems, we developed a new approach to measure P_dc_. More specifically, our goal was to ensure that the protocol is of short duration, suitable for spot fluorometers (i.e., fluorometers without axial scanning), and ideal for measurements with fluorometers of lower fluorescence sensitivity. To enable fluorescence detection at a high SNR while maintaining high axial resolution, we chose to administer multiple drops of fluorescein, so that increased accumulation of the dye in the stroma could be achieved. While multiple drops produced measurable levels of fluorescence from the stroma, a new approach for the calculation of P_dc_ had to be developed. In addition, the propsed multi-drop protocol avoids concentration quenching during all fluorescence recordings. Our measurements using the multi-drop method in a cohort of healthy subjects have revealed that the P_dc_ is at least 11-fold higher than previously reported values based on the single-drop method [37]. Overall, it is expected that our efforts will not only enhance understanding of the epithelial pathophysiology, but will also enable quantitative assessments of the pharmacokinetics of topical drug delivery.

## Materials and methods

### Subjects

Healthy subjects between 20 and 46 years of age (28 ± 8.1 years; n = 29) were recruited. There were 15 males of age 20–33 years (27 ± 3.8 years) and 14 females of age 20–46 years (27 ± 6.6 years). Subjects with past or present ocular diseases and diabetes were excluded. Subjects on contact lenses, with signs and symptoms of dry eye disease, or on topical medications were also excluded. Subjects using artificial tears were also excluded. Informed consent was obtained from all subjects before any measurements were undertaken. Most of the subjects were visitors or employees at the eye hospital (SankaraNethralaya, Chennai, India). The study protocol adhered to the tenets of the Declaration of Helsinki and approved by the Institutional Review Board at the eye hospital (Medical Research Foundation; Chennai, India).

### Custom-made ocular spot fluorometer

A standard slit lamp was adapted to measure fluorescence from any desired spot in the anterior segment of the eye without axial scanning. An adjustable slit for the emitted light (referred to as the emission/collection slit) was placed confocal to the projection slit (a.k.a., the excitation/illumination slit) of the slit lamp to enable depth-resolved measurements. The size of the excitation slit was 4 mm x 0.5 mm for measurements in both the stroma and tear film. The emission slit was made equal to or slightly smaller than the excitation slit. For rejecting electronic noise as well as interference from the ambient light, we employed the principle of lock-in amplification for the detection of the emission. Accordingly, the excitation light intensity was modulated as a sine wave. This was achieved by replacing the halogen lamp of the slit-lamp with a high power white LED (10 Watts). The LED was modulated at 10 kHz using a custom-made LED driver (a linear amplifier with a bandwidth of 200 kHz), which was coupled to a standard function generator (Stanford Research Systems Inc., Model 345). The LED output was passed through a cobalt-blue filter to obtain the modulated excitation for fluorescein. Light through the emission/collection slit was passed through a barrier filter (530 ± 10 nm) and then detected by a photomultiplier tube (PMT; R928HA; Hamamatsu, Inc). The output of the PMT and the sync output of the function generator were fed to the signal and reference inputs of a lock-in amplifier (Model 7260, Signal Recovery Inc., USA), respectively. The fluorescence measurements were recorded on the PC through a USB-GPIB interface at 100 Hz and were averaged further to minimize the noise. Specifically, the tear film and stromal fluorescence at each time point consisted of 15–20 samples taken over a period of 4 seconds. Each sample, in turn, was an average of more than 30–50 samples.

### Epithelial permeability to fluorescein by the multi-drop method

**Fig 1:**
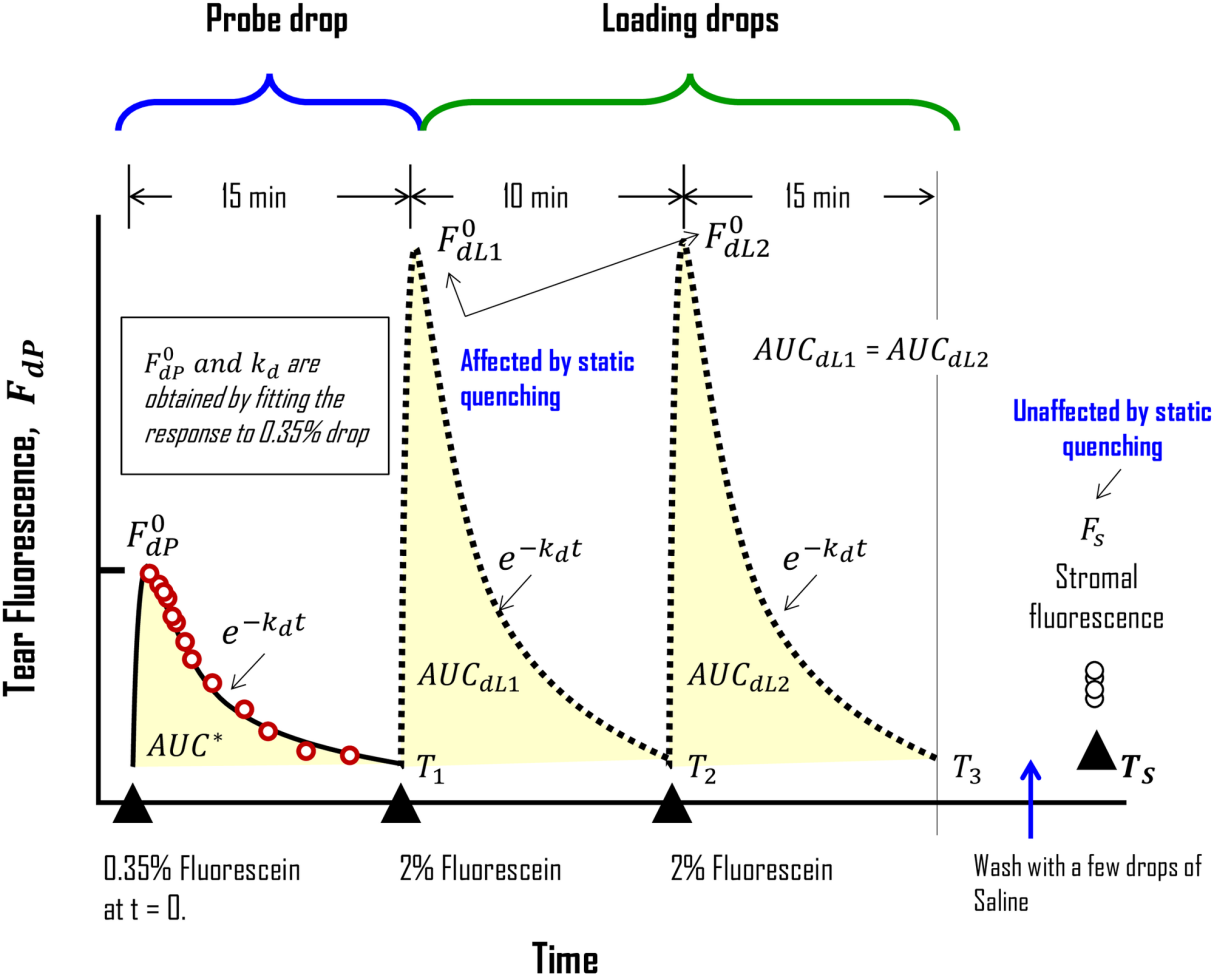
Schematic of the multi-drop protocol for the measurement of epithelial permeability. At t = 0, a 0.35% fluorescein drop (0.35 gm of fluorescein/100 mL PBS buffer) is instilled on the bulbar conjunctiva and the tear fluorescence is measured (shown by red unfilled circles). After clearance of the dye (usually < 15 min), two drops of 2% fluorescein (2 gm of fluorescein/100 mL PBS buffer) are instilled 10 min apart (T_1_ and T_2_). About fifteen minutes after the second drop, the ocular surface is washed with CMC solution (carboxymethyl cellulose solution; Blue arrow). Next, stromal fluorescence is measured 3–4 times at time T_s_ (usually within 5–10 min after T_3_). AUC_dL1_ and AUC_dL2_, which are assumed to be equal, are estimated based on the area under the curve calculated for the 0.35% drop (AUC*). The tear fluorescence in response to the probe drop is fitted to a single-exponential decay to determine F^0^_dp_ and k_d_. F^0^_dp_ is then used to estimate F^0^_dL1_ and F^0^_dL2_. k_d_ for the 2% drops is assumed to be the same as that for the 0.35% drop. Hence, the first 0.35% drop is referred to as the probe drop. The 2% drops have been employed to load the stroma with measurable levels of fluorescein so that noise-free measurements of the stromal accumulation can be obtained. Therefore, the 2% drops are referred to as the loading drops.

We first provide an overview of the new multi-drop method and give details subsequently using Fig 1. First, a drop of low concentration fluorescein (referred to as the probe drop) is instilled, and the dynamics of its clearance from the ocular surface is measured using the fluorometer. Next, two loading drops containing a high concentration of fluorescein are instilled sequentially ~10 min apart, and the accumulation of the dye in the stroma is measured ~ 15 min after the second drop. These measurements along with the stromal thickness are then used to determine the corneal epithelial permeability to fluorescein (P_dc_).

The concentration of fluorescein in the probe drop is small, and hence measurements of fluorescence from the tear film is devoid of concentration quenching. The driving force for the penetration of fluorescein into the stroma following the loading drops is estimated based on the dynamics of fluorescein clearance from the ocular surface for the probe drop. In other words, we assume that the fluorescein clearance of the loading drops from the ocular surface is equal to that of the probe drop. We use loading drops of high concentration to ensure higher accumulation of the dye in the stroma, which allows for measurements with a high signal-to-noise ratio (SNR). Since the accumulation of the dye in the stroma is usually very small even after the loading drops, the high concentration of the dye in the loading drops does not cause concentration quenching in the measurements of stromal fluorescence. Finally, based on the principles similar to those employed for the calculation of P_dc_ in the single-drop method, we have derived new equations to compute P_dc_ from measurements obtained with the multi-drop protocol.

Sodium fluorescein was obtained as a sterile 20% solution (Medimark Agencies, India). It did not contain benzalkonium chloride or other preservatives. Aliquots of topical drops were prepared quantitatively in sterile PBS as needed. All subjects underwent a general eye examination before experiments during which we recorded their central corneal thickness (CCT) by anterior segment OCT (SS-1000, Casia Tomey). Also, we measured autofluorescence from the corneal stroma. The fluorescence from the tears in the absence of fluorescein was negligible, and hence, autofluorescence from the tears was assumed zero. Subsequently, subjects underwent P_dc_ measurements as per the multi-drop protocol depicted by the timeline in Fig 1.

As per the protocol, we first instilled a 2-μL drop of 0.35% fluorescein (probe drop) on the superior bulbar conjunctiva. The subjects were asked to blink a few times to ensure uniform spreading of the dye over the pre-corneal tear film. After 3–4 quick blinks, the clearance of fluorescein from the tears was measured by recording tear fluorescence every 15 seconds for the first 2 minutes and every 30 seconds after that until changes in the measured fluorescence became negligible. Subsequently, two 6 μL drops of 2% fluorescein (loading drops) were administered 10 min apart. After 15 min of the second loading drop, the ocular surface and the fornices were washed using pre-formulated and sterile carboxymethyl cellulose (CMC) solution devoid of benzalkonium chloride or other preservatives. After the wash, fluorescence from the stroma was recorded 15–20 times and averaged to obtain the uncorrected stromal fluorescence (uF_s_). The corrected stromal fluorescence (F_s_), which is indicative of accumulated fluorescein in the stroma, was obtained by subtracting the autofluorescence from uF_s_. Although two loading drops were used, staining of the corneal epithelium was not noticed in any of the experiments (49 eyes of 29 subjects). This is consistent with the protocol employed for the measurement of corneal endothelial permeability wherein instillation of more than 7 drops of 5% fluorescein is typically employed [44, 64, 65].

Measured central corneal thickness (CCT), fluorescence clearance kinetics during the probe drop, and F_s_ were used to calculate P_dc_ as outlined below. All measurements for the estimated P_dc_ were performed by two examiners to avoid potential inter-observer variabilities.

### Statistical analysis

The tear fluorescence curves were fitted to a single exponential decay by a non-linear least squares analysis using Prism 5.0 (GraphPad^™^ Software, Inc USA). The fitting to the decay was constrained to a steady state value of zero, consistent with autofluorescence of the tears being negligible. The comparison of the means of k_d_ for 2 and 6 μL drops in four different subjects was also performed with GraphPad^™^ based on Mann-Whitney U tests. The Monte Carlo Simulations (MCS) [66–68] (detailed in S1 Appendix), employed to evaluate the robustness of the experimental P_dc_ estimate, were carried out by a custom-made software program in LabVIEW (National Instruments Inc, Austin, TX).

## Results

### Linearity, sensitivity, and axial resolution of the spot fluorometer

**Fig 2:**
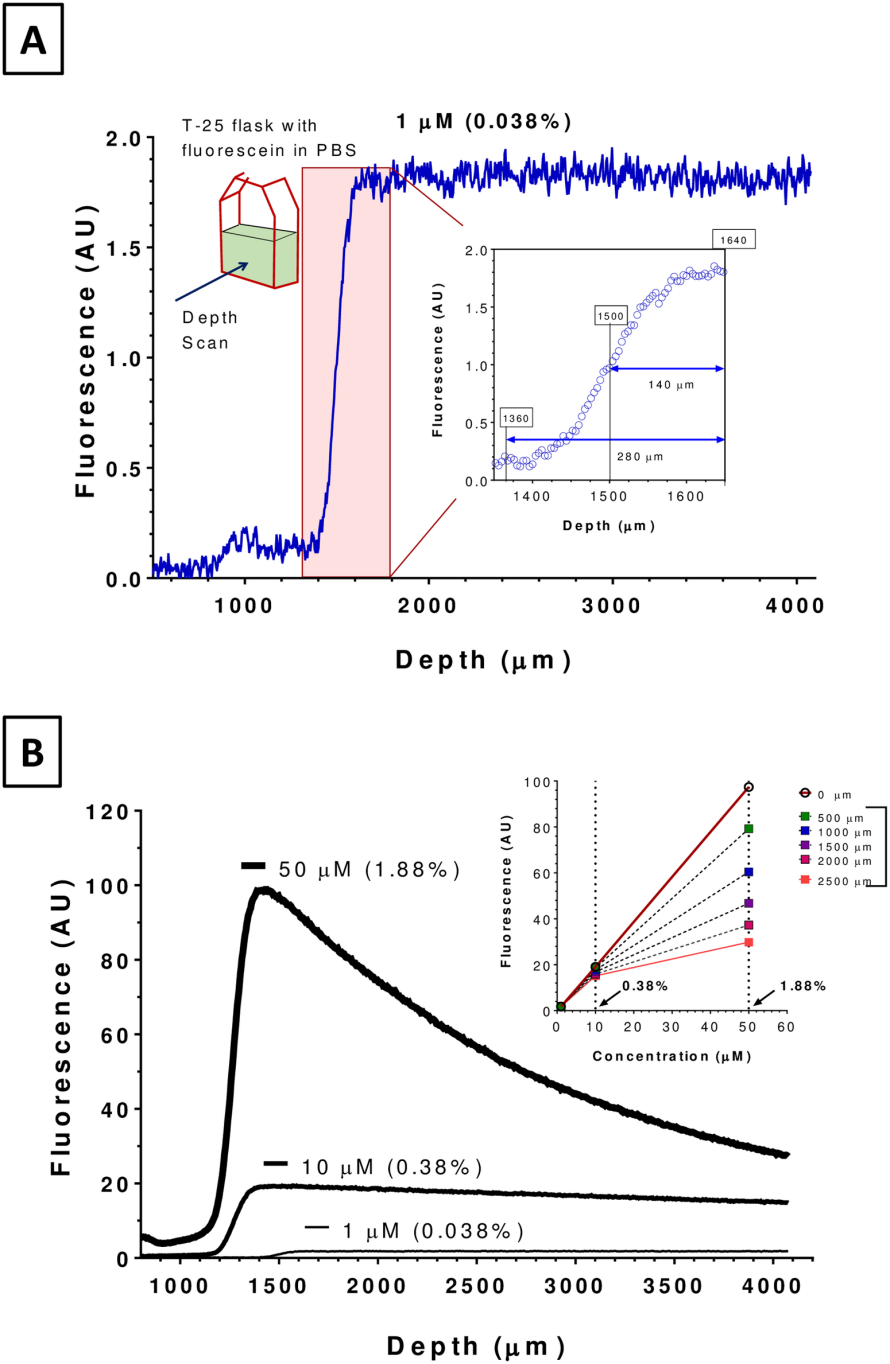
Linearity, depth resolution, and concentration quenching. Three solutions of fluorescein, prepared with PBS at 1, 10, and 50 μM (corresponding to 0.038%, 0.38%, and 1.88% (w/v), respectively) were contained in T-25 flasks. Depth-resolved fluorescence measurements were then obtained by positioning the flask along with the optical axis of the fluorometer. The angle between the excitation and the emission arms was held at 45°. Concentrations of fluorescein in % (provided in parenthesis) are in w/v basis. **Panel A**: Typical fluorescence *vs*. depth profile obtained with 1 μM solution. Note that the fluorescence of the solution remains constant over the depth of the scan. The fluorescence change from background to the new plateau occurs over a transition depth ~ 280 μm (inset on the right). This is a measure of the axial resolution of the instrument, which can also be specified as full-width at half maximum (FWHM; 140 *μ*m). **Panel B**: Fluorescence *vs*. depth profiles obtained with fluorescein at 1, 10, and 50 μM solutions. Unlike the fluorescence profile for 1 μM solution (thin line), the fluorescence for 50 μM (thick line) decreases with increasing depth, indicating concentration quenching. The fluorescence *vs*. depth profile for the 10 μM solution (0.38%) also shows concentration quenching, but it is marginal. Hence, the concentration of fluorescein in the probe drop was kept below 0.38% in all our experiments. The inset in Panel B summarizes the concentration quenching at different depths for the three different solutions.

We first assessed the linearity, sensitivity, and axial resolution of the custom-made spot fluorometer. These factors would be critical to assess the precision, reproducibility, and reliability of P_dc_ measurements. The investigations were carried out with fluorescein diluted in PBS at a pH of 7.4 contained in T-25 flasks. The flasks were held on a linear stage (coupled to a stepper motor) located at the chin rest of the fluorometer. Axial scans across the solution were performed under computer control (inset of Fig 2A; Top Left). Fig 2A shows a typical fluorescence depth profile (blue line) with 1 μM fluorescein (i.e., 0.038% w/v of fluorescein). A steady level of fluorescence across the scanned depth shows the absence of concentration quenching. The measured fluorescence has a SNR (i.e., mean/SD of the signal) of 44, indicating a high measurement sensitivity. Fig 2B presents fluorescence *vs*. depth profiles at higher concentrations. At 50 μM (1.88% of fluorescein), a rapid decline in the measured fluorescence is observed with increasing depth. At 10 μM (0.38% fluorescein), a smaller decrease in the measured fluorescence with depth is noted. These findings suggest that concentration quenching occurs at concentrations > 10 μM. Based on these observations, we chose the concentration of the probe drop to be 0.35% for all our experiments. Thus, we ensured that none of our measurements is affected by non-linearity due to concentration quenching [29, 62].

The measurements in Fig 2A also highlight the depth resolution of the instrument. Specifically, the plot in the inset (unfilled circles; right side insert) indicates that the rise in fluorescence at the transition point from the flask into the fluorescein solution occurs over a depth of 280 μm (i.e., 1640 μm -1360 μm). In other words, the depth of the focal diamond formed by the intersection of the excitation and emission beams is ~ 280 μm. This axial resolution is better than what is known for Fluorotron Master^™^ (> 0.5 mm) [56, 69, 70].

**Fig 3:**
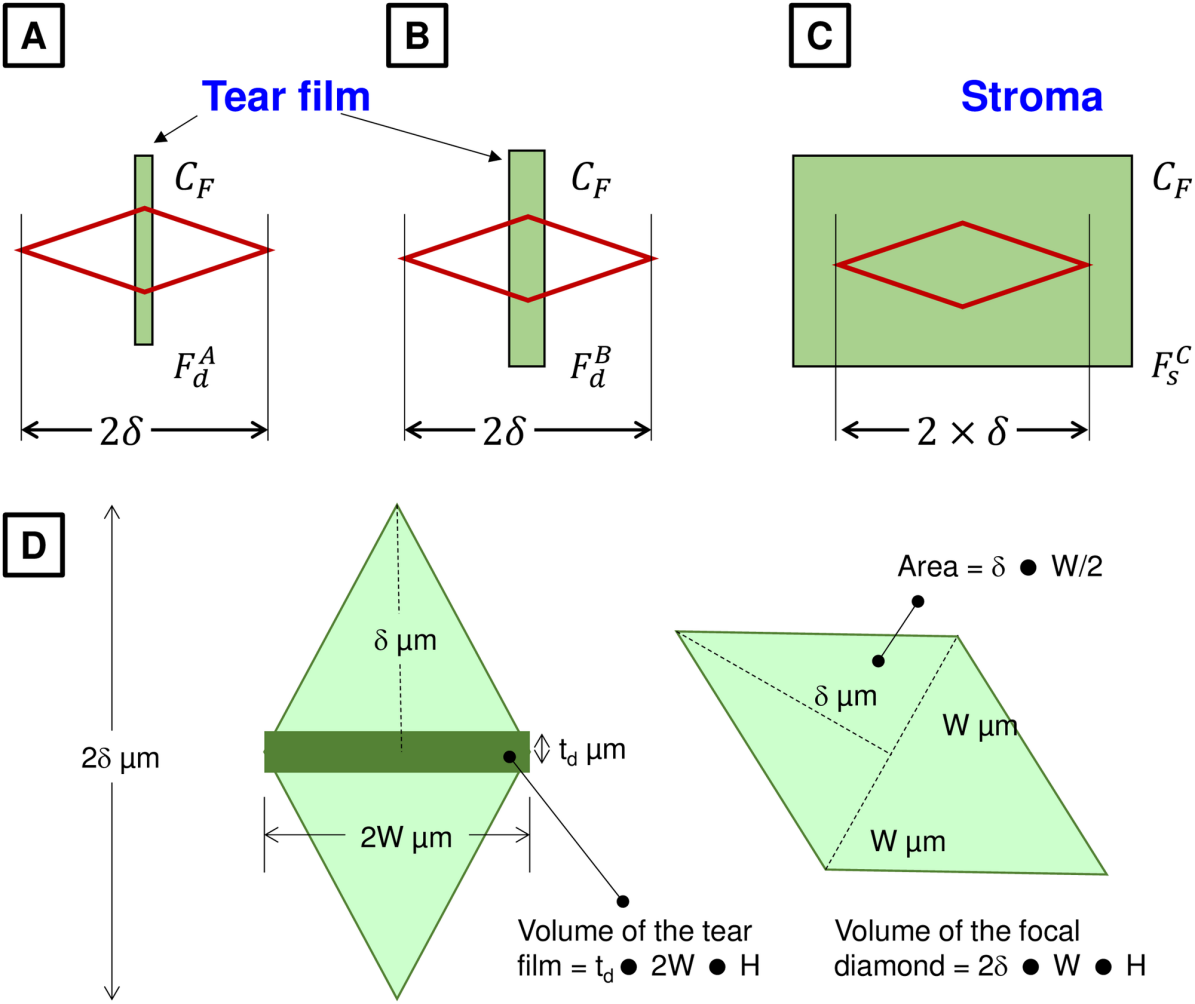
Effect of the axial resolution on the measured fluorescence. The depth of the focal diamond is 2δ. From Fig 2, we note that δ is 140 μm. **Panel A**: Focal diamond (RED lines) across the tear film with fluorescein. Note that the tear film does not span the entire depth of the focal diamond. In the figure, F^A^_d_ and C_F_ refer to the measured tear fluorescence and concentration of fluorescein in the tear film, respectively. **Panel B**: Tear film is thicker compared to that in Panel A so that F^B^_d_ >F^A^_d_. **Panel C**: Focal diamond is positioned in the stroma. In this case, the measured fluorescence F^C^_S_ would be proportional to C_F_ but independent of the stromal thickness, which is greater than 2δ. **Panel D**: Estimation of ICF. The intersection of the tear film with the focal diamond is a parallelepiped of volume given by t_d_ x 2W x H, where t_d_ is the thickness of the tear film, 2W is the maximum width of the focal diamond, and H is the thickness of the excitation slit beam. The volume of the focal diamond is given by 2δ x W x H, where 2δ is the axial resolution of the instrument (2δ ~ 280 μm). Hence, the ratio of the volume of the focal diamond to that of volume of intersection between tear film and focal diamond (defined as ICF, instrument correction factor) is given by (2δ x W x H) / (t_d_ x 2W x H). Hence, ICF would be δ/t_d_, which is equal to 47 assuming a tear film thickness of ~ 3 μm [38, 41–43]. This estimation assumes that the fluorescence in the stroma for a given concentration of fluorescein is not different from an equivalent amount of fluorescein in water. More specifically, fluorescein is assumed unbound in the stroma.

The potential impact of the depth of the focal diamond on the measured value of fluorescence in the stroma and tears is illustrated in Fig 3. For measurements in tears, it is evident that the tear film does not span the depth of the focal diamond (Fig 3B). Moreover, since the stromal thickness is greater than the depth of the focal diamond, the measured fluorescence from the stroma is only proportional to the concentration of the dye in the stroma assuming that fluorescein does not bind to components of the stroma (Fig 3C). Since the tear film thickness (t_d_) is smaller than the axial resolution of the instrument (δ), the ratio of the intersection volume of the focal diamond with the stroma to that with the tear film can be approximated by δ/t_d_. We refer to this ratio as instrument correction factor or ICF (Fig 3). Assuming a tear film thickness of 3 μm [38, 41–43] and recalling 2δ = 280 μm, we assert that for our custom-made fluorometer, ICF is ~ 47. For a given concentration of fluorescein, the fluorescence measured in the tear film should be multiplied by ICF to predict the fluorescence from the stroma at the same concentration.

### Dynamics of fluorescein clearance following the probe drop

For instilled drops of small volume V^0^_i_, such as the probe drop (2 μL; 0.35%), we assume the tear clearance of fluorescein to follow a single exponential decay [6, 71]. Therefore, the concentration of the dye in the tears at any time t (denoted by C_dP_ (t)) after instillation of the probe drop (Fig 1) can be written as

$$C_{dP}(t)=C_{dP}^{0}e^{-k_{d}t}$$ (1)

Where C^0^_dP_ is the concentration of fluorescein in the tears at time t = 0. Table 3 below highlights all the mathematical notations.

**Table 3:** Mathematical notations.

Symbol	Description	Units
V_i_	Instilled volume on the ocular surface	μL
C_dP_	Concentration of the dye in the tears at any time t after the probe drop	μM
C^0^_dp_	Concentration of dye in the tears immediately (t = 0) after the probe drop	μM
k_d_	Elimination rate constant	sec^-1^
t^d^_1/2_	Half-life of the dye in tears = 0.693/k_d_	sec
F_dP_	Tear fluorescence after the probe drop	mV
α	Proportionality constant for the calibration curve between tear fluorescence and concentration of the dye in the tears	μM /mV
F^0^_dP_	Tear fluorescence immediately (t = 0) after the probe drop	mV
AUC*	Area under the fluorescence curve following the probe drop	mV sec
P_dc_	Corneal epithelial permeability to fluorescein	nm/sec
A	Surface area of the cornea	mm^2^
m_s_	Mass of fluorescein in the stroma	μg
Q	Mean stromal thickness	μm
C_dL1_	Concentration of the dye in tears following loading drop 1	μM
C_s_	Concentrations of the dye in the stroma	μM
F_s_	Stromal fluorescence	mV
β	Proportionality constant for the calibration curve between tear fluorescence and concentration of the dye in the stroma	μM /mV
AUC_dL1_	Area under the fluorescence curve following the first loading drop	mV sec
AUC_dL2_	Area under the fluorescence curve following the second loading drop	mV sec
AUC_dL_	[AUC_dL2_] = [AUC_dL1_] = [AUC_dL_]	mV sec
M_p_	Mass of the dye in the probe drop	μg
V_d_	Volumes of tears	μL
M_L_	Mass of the dye in the loading drop	μg
C^0^_dP_	Concentration of the dye in tears immediately following the probe drop	μM
C^0^_dL_	Concentration of the dye in tears immediately following the loading drop	μM
CCT	Central corneal thickness	μm

C^0^_dP_ is dependent on the tear volume, instilled drop volume, and the concentration of fluorescein in the instilled drop. k_d_ is the elimination rate constant (with units of per min) which is influenced mainly by the blink frequency, tear secretion rate, tear outflow rate, tear evaporation, and the volume of tears.

Based on Eq 1, the half-life for fluorescein clearance can be given by t^d^_1/2_ = 0.693/ k_d_ and is found to be 2–4 min in healthy eyes [44, 60]. Since the concentration in the instilled drop (0.35%) is less than the threshold for the onset of concentration quenching (< 0.38% as per Fig 2B), we assume a linear relationship between the concentration in the tears and the measured tear fluorescence:

$$C_{dP}(t)=\alpha F_{dP}$$ (2)

Where α is the proportionality constant between tear fluorescence (F_dP_) and fluorescein concentration (C_dP_ (t)). Applying Eq 2 at time t = 0,

$$C_{dP}^{0}=\alpha F_{dP}^{0}$$ (3)

As shown in Fig 3A, since the depth of the focal diamond is much larger than the thickness of the tear film, α would be dependent on the axial resolution of the instrument. Substituting Eqs 2 and 3 in Eq 1, we get

$$F_{dP}(t)=F_{dP}^{0}e^{-k_{d}t}$$ (4)

Integrating both sides of Eq 4 with respect to time (between 0 and time T), we get

$$\left[{AUC}_{P}]=F_{dP}^{0}\int_{0}^{T}e^{-k_{d}t}\;dt=\frac{F_{dP}^{0}}{k_{d}}(1-e^{-k_{d}T}\right)$$ (5)

where [AUC_P_] = ᶴ_0_^T^ F_dP_ dt is the area under the tear fluorescence curve following the probe drop.

For large T (5 x t^d^_1/2_or i.e., >10 min), Eq 5 above simplifies to

$$[{AUC}_{P}]=\frac{F_{dP}^{0}}{k_{d}}$$ (6)

**Fig 4:**
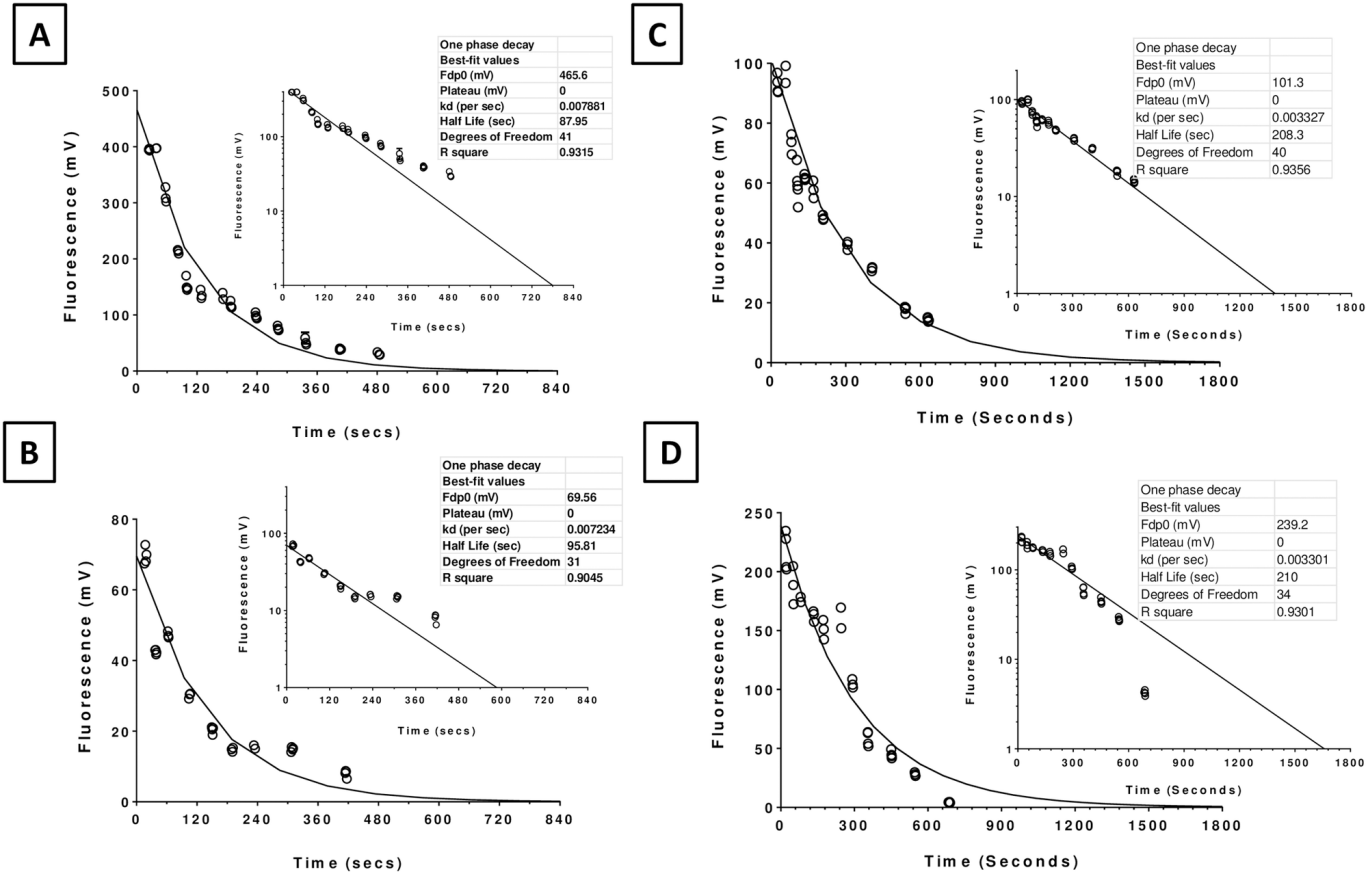
Inter-subject variability of fluorescein clearance after the probe drop in two subjects. The excitation slit was focused on the tear film and the fluorescence *vs*. time profile was obtained for 10 min. F^0^_dP_ and k_d_ are the intercept and slope, respectively, of the fluorescence decay in the tear film as per the exponential decay curve (Eq 4) using non-linear least squares. Half-lives t^d^_1/2_ indicated in the inset were calculated from k_d_. **Panels A and B**: Data from a subject showing rapid clearance of fluorescein with half-lives of only 88 and 96 seconds in the left and right eyes, respectively. **Panels C and D**: Data from a different subject showing a relatively slower clearance with half-lives of 208 and 210 seconds in the left and right eyes, respectively.

**Fig 5:**
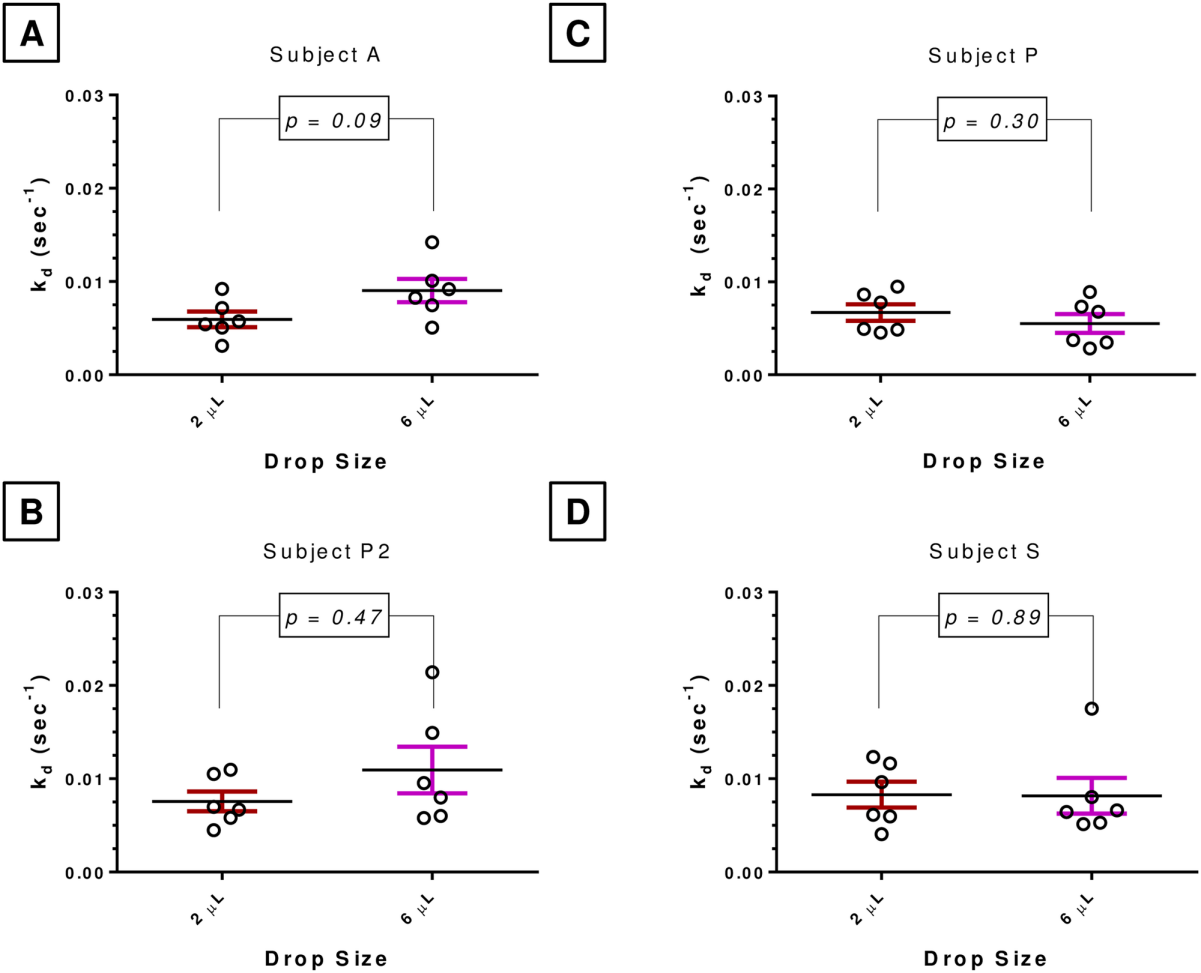
Effect of instilled volume on fluorescein clearance (k_d_) in four different subjects. The data also shows the intra-subject variability of fluorescein clearance in repeat trials. **Panels A-D** show k_d_ estimated from independent probe drops of fluorescein (0.35%) at 2 or 6 μL each dropped in a single subject ~15 min apart. Paired t-tests show lack of any significant difference between the means of k_d_ measured at the two instilled volumes.

As mentioned earlier, concentration quenching is not observed at 0.35% of fluorescein (Fig 2B) [29, 62]. Therefore, the probe drop allows for the estimation of k_d_ and F^0^_dP_ in Eq 6 without any influence of concentration quenching. Both parameters can be estimated by fitting Eq 1 to tear fluorescence *vs*. time data after the probe drop. Fig 4 shows the variability of the tear fluorescence dynamics between subjects. The repeatability in a given subject of the tear-fluorescence decay profiles is presented in Fig 5.

### Fluorescein accumulation in the stroma after the loading drops

The loading drops contain 2% fluorescein (Fig 1). Therefore, accumulation of fluorescein in the stroma would be significant when compared to the accumulation following probe drop, which was negligible in our experiments. Increased accumulation will allow us to precisely measure the stromal fluorescence, and hence accurately estimate P_dc_. In the following, we relate P_dc_ quantitatively to the accumulation of fluorescein in the stroma in response to the two loading drops. First, we define corneal epithelial permeability to fluorescein (P_dc_) by the following phenomenological equation:

$$\frac{dm_{s}}{dt}≝P_{dc}\;A\;(C_{dL1}-C_{s}),\;where\;m_{s}=Q\;A{\;C}_{s}$$ (7)

In Eq 7, m_s_ is the mass of fluorescein in the stroma, A is the surface area of the cornea, and Q is the mean stromal thickness. (Q x A) is the stromal volume. C_S_ and C_d_ refer to concentrations of fluorescein in the stroma and tears, respectively. Specifically, C_dL1_ is the concentration of fluorescein in the tears following the first loading drop. Substituting for m_s_ and assuming C_S_<< C_dL1_ at all times, we can rewrite Eq 7 as

$$Q\frac{dC_{s}}{dt}=P_{dc}C_{dL1}$$ (8)

Since the accumulated fluorescein in the stroma is within the limits of concentration quenching (<< 0.35% fluorescein; measured stromal concentration following the loading drop 1), we again assume linearity between measured F_S_ and the stromal concentration C_S_. Thus,

$$C_{s}(t)=\beta F_{s}$$ (9)

Where β is the proportionality constant between the measured stromal fluorescence F_s_ and the concentration of fluorescein in the stroma (C_S_ (t)).

Applying Eqs 2 and 9, we can rewrite Eq 8 as

$$\beta Q\frac{dF_{s}}{dt}=P_{dc}{\alpha F}_{dL1}$$ (10)

We note that the calibration constants α and β are not identical because the depth of the focal diamond is larger than tear film (~ 3 μm) but smaller than the stromal thickness (~ 480 μm; Fig 3).

Integrating both sides of Eq 10 with respect to time between T_1_ and T_2_ (as specified in Fig 1), we get

$$\beta Q\;[F_{s}(T_{2})-\;F_{s}(T_{1})]=P_{dc}\;\alpha \;[{AUC}_{dL1}]$$ (11)

where [AUC_dL1_] = ᶴ_T1_^T2^ F_dL1_ dt is the area under the fluorescence curve following the first loading drop.

Since we administer another drop at T_2_ (Fig 1), similar to Eq 11, we can write

$$\beta Q\;[F_{s}(T_{3})-\;F_{s}(T_{2})]=P_{dc}\;\alpha \;[{AUC}_{dL2}]$$ (12)

where [AUC_dL2_] = ᶴ_T2_^T3^ F_dL2_ dt is the area under the fluorescence curve following the second loading drop.

Adding Eqs 11 and 12, we get

$$\beta Q[F_{s}(T_{2})-\;F_{s}(T_{1})+\;F_{s}(T_{3})-\;F_{s}(T_{2})]=P_{dc}\alpha ([{AUC}_{dL1}]\;+[{AUC}_{dL2}]\;)$$ (13)

Since the two loading drops are identical at T_1_ and T_2_ (volumes and location on the bulbar conjunctiva), as a first approximation, we claim

$$\left[{AUC}_{dL2}]=[{AUC}_{dL1}]=[{AUC}_{dL}\right]$$

This simplifies Eq 13 to

$$\beta Q[-\;F_{s}(T_{1})+\;F_{s}(T_{3})]=2\;P_{dc}\alpha [{AUC}_{dL}]$$ (14)

Since fluorescein in the stroma before the first loading drop at T_1_ is negligibly small (F_s_(T_1_)~ 0), Eq 14 implies that

$$P_{dc}=\frac{Q\;\beta F_{s}(T_{3})}{2\;\alpha [{AUC}_{dL}]}$$ (15)

where F_s_(T_3_) is the average stromal fluorescence measured ~ 15 min after instillation of the second drop (i.e., at T_s_ in Fig 1). In general, α is the proportionality constant for F_d_ *vs*. C_d_ so that C_d_ = α x F_d_. Also, β is the proportionality constant for F_s_ *vs*. C_s_ so that C_s_ = α x F_s_. Since the tear film thickness is less than that of the focal diamond, α<β. We also note that Eq 15 is valid for small T_3_ (< 30 min) so that the loss of fluorescein from stroma across the endothelium remains negligible.

### Calculation of the epithelial permeability

As per Eq 15, we need to know Q,α, β, F_s_(T_3_) and [AUC_dL_] for calculating P_dc_ after instillation of two loading drops of 2% fluorescein. The value of [AUC_dL_] can be estimated based on the dynamics of fluorescein clearance of the probe drop. We estimate from the probe drop because any tear fluorescence measurements after the loading drops (2% fluorescein) would be confounded by concentration quenching, for which the threshold is 0.38% (Fig 2). For the estimation of [AUC_dL_], we define V_i_ and V_d_ as the volumes of the instilled drop and the tears, respectively. Further, we define M_P_ as the mass of fluorescein in the probe drop. Then, we can write the following equation to determine the concentration of fluorescein immediately after the instillation of the probe drop and subsequent mixing with the tears:

$$C_{dP}^{0}=\frac{M_{P}}{V_{i}\;+\;V_{d}}$$ (16)

This equation assumes no spillover of the tears following the probe drop (2 μL) and complete mixing. Similarly, if M_P_ denotes the mass of fluorescein in the loading drops (6 μL), we can write the following equation for the concentration of fluorescein immediately after the instillation of the loading drops and their complete mixing in the tears:

$$C_{dL}^{0}=\frac{M_{L}}{{Vi+V}_{d}}$$ (17)

Assuming C^0^_dP_ = α F^0^_dP_, fluorescence from the 2 μL probe drop at time t = 0 can be written from Eq 16 as

$$F_{dP}^{0}=\frac{M_{P}}{\alpha \;(2+V_{d})}$$ (18)

where F^0^_dP_ represents the tear fluorescence at t = 0 following the probe drop.

Similarly, for 6-μL loading drops, fluorescence at time t = 0 can be written from Eq 17 as

$$F_{dL}^{0}=\frac{M_{L}}{\alpha \;(6+V_{d})}$$ (19)

Dividing Eq 19 by 18, we get

$$\frac{F_{dL}^{0}}{F_{dP}^{0}}=\frac{M_{L}}{M_{P}}\frac{{(2+V}_{d})}{{(6+V}_{d})}$$ (20)

Now, similar to Eq 6, we can calculate [AUC_dL_] as

$$[{AUC}_{dL}]=\frac{F_{dL}^{0}}{k_{d}}$$

Substituting for F^0^_dL_ in Eq 20, we get

$$[{AUC}_{dL}]=F_{dP}^{0}\frac{1}{k_{d}}\frac{M_{L}}{M_{P}}\frac{{(2+V}_{d})}{{(6+V}_{d})}$$ (21)

In other words, [AUC_dL_] necessary for calculating P_dc_ can be obtained from F^0^_dP_ and k_d_, which are determined by the instillation of the probe drop. Hence, for the two-drop protocol, we calculate P_dc_ based on Eqs 15 and 21 by

$$P_{dc}=\frac{k_{d}Q\beta F_{s}(T_{3})}{2\;\alpha \;F_{dP}^{0}}\frac{M_{P}}{M_{L}}\frac{{(6+V}_{d})}{{(2+V}_{d})}$$ (22)

Thus, assuming a value for V_d_, we can calculate P_dc_ knowing Q,α,β, F_s_ (T_3_), k_d_ and F^0^_dP_. M_P_ and M_P_ are fixed at 0.35% and 2% for probe and loading drops, respectively. The mean stromal thickness (Q) was calculated as [(1.06 x CCT)– 50], where CCT is the central corneal thickness as measured by OCT in our experiments. We have assumed epithelial thickness to be 50 μm on average. The formula for the average stromal thickness was obtained from 68 eyes in independent experiments based on OCT measurements. Referring to Eqs 3 and 9, we also note that β/α in Eq 22 is equal to ICF (~47 for our instrument).

### Measurement of permeability

As shown in the timeline for the protocol (Fig 1), our first step was to assess dynamics of fluorescein clearance in a given subject by a probe drop. The data was then used to estimate k_d_ and F^0^_dP_ in order to calculate P_dc_ by Eq 22. As noted before, Fig 4 shows the typical profiles of fluorescence decay following the probe drop. The estimation of F^0^_dP_ and the curve fit to tear fluorescence data requires measurement of tear fluorescence immediately after the administration of the probe drop. In most subjects, the first measurement could be made in less than ~ 20 seconds after administering the probe drop. The tear fluorescence data invariably showed single exponential decay kinetics as expected. These could be fitted to Eq 1 with the correlation coefficient typically > 0.9. Since the tear fluorescence before the probe drop is negligible, the fitting of tear fluorescence *vs*. time to single exponential decay kinetics was constrained to a steady state value of zero.

**Fig 6:**
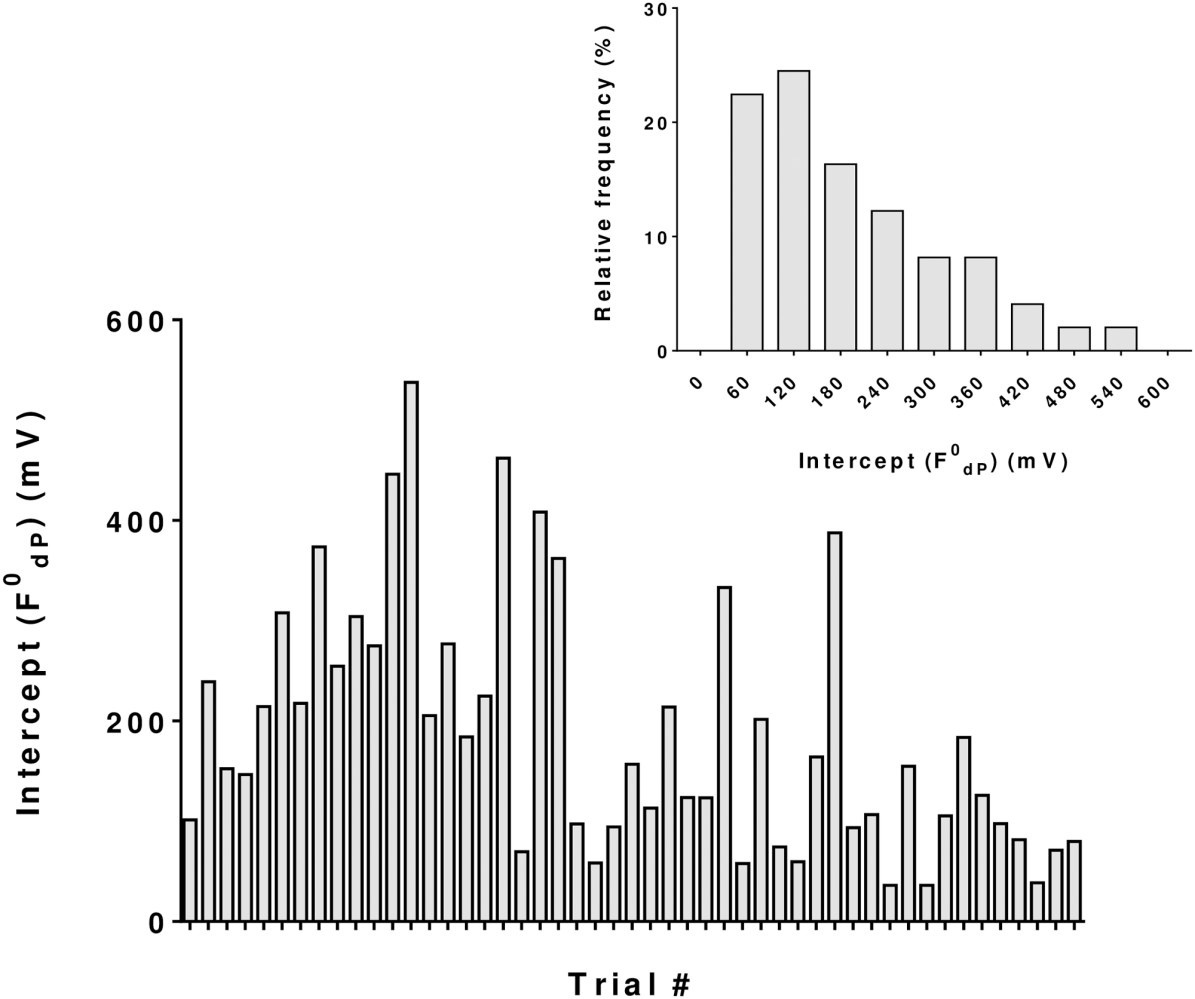
Estimated tear fluorescence at time t = 0 after instillation of the probe drop (F^0^_dP,_). The Y intercept (tear fluorescence at t = 0, F^0^_dP_) of the fluorescence *vs*. time plot was obtained by fitting data to the exponential decay as per Eq 4: ln F_dP_ (t) = ln F^0^dP—k_d_ t. The mean and SD values are 206.51 and 113.89 mV, respectively (n = 49 eyes and 29 subjects).

**Fig 7:**
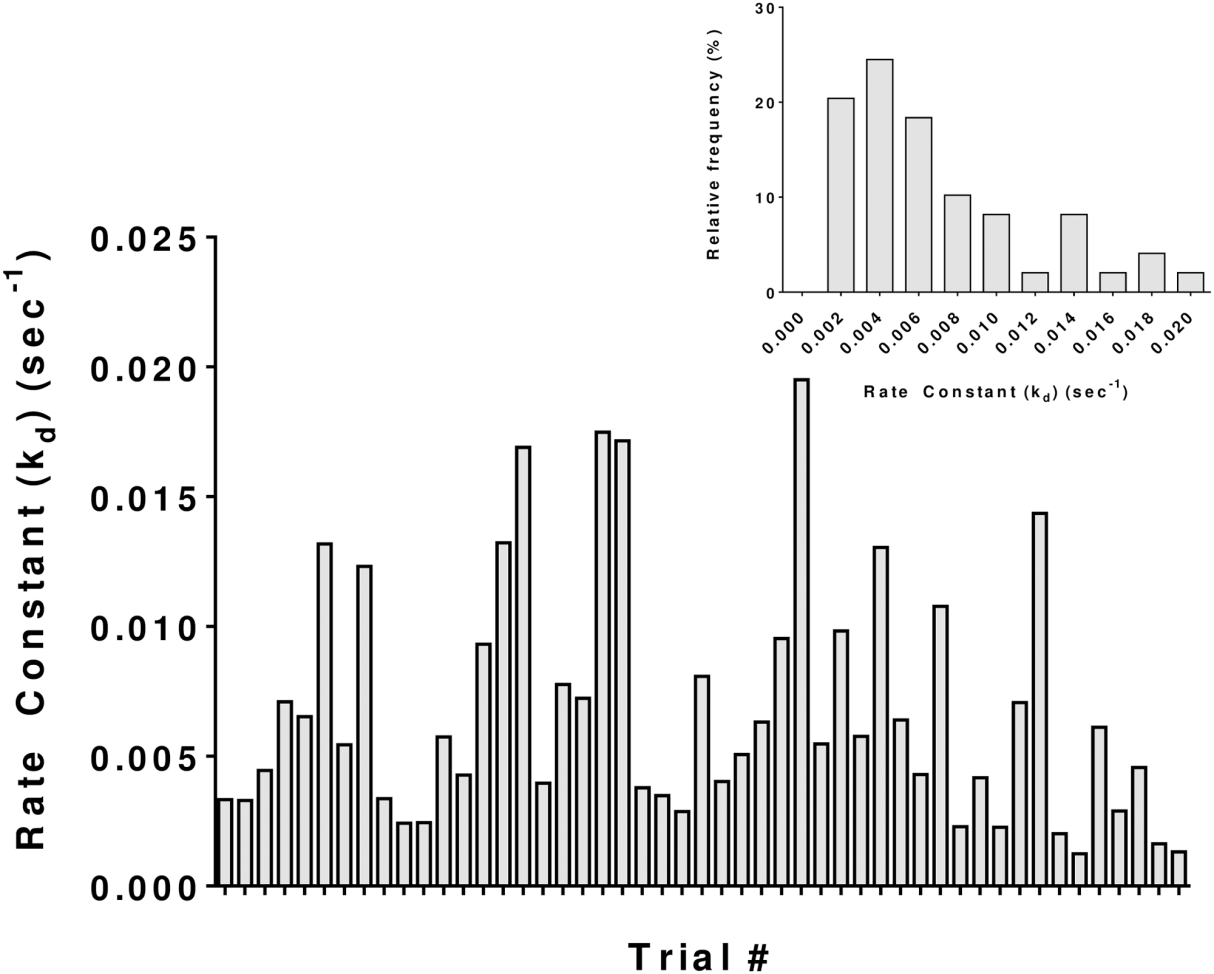
Estimated fluorescein elimination rate constant (k_d_) following instillation of the probe drop. k_d_ was obtained as the slope of the fluorescence decay by fitting to Eq 4 according to ln F_dP_ (t) = ln F^0^dP—k_d_ t. The mean and SD values are 0.0142 and 0.0107 sec^-1^, respectively (n = 49 eyes and 29 subjects).

The fluorescence at time t = 0 (F^0^_dP_) which is dependent on the tear volume, varied by ~ 9-fold (206 ± 113.9; 49 eyes of 29 subjects), as depicted in Fig 6. Similarly, in agreement with variations in the tear flow rate, blink frequency, and tear volume, k_d_ varied from 0.0015 sec^-1^ to 0.044 sec^-1^ (0.014 ± 0.010 sec^-1^; 49 eyes of 29 subjects) (Fig 7). This corresponds to a variation in the half-life from 53 seconds to 210 seconds (Fig 4).

At 15 minutes following the probe drop, the stromal fluorescence (F_s_ (T1)) did not increase significantly beyond the baseline (p < 0.05). However, after the two loading drops, F_s_ (T3) (denoted as F_s_ (Ts)) increased by 10-fold compared to the background (p < 0.05; 10.19 ± 8.22). Moreover, F_s_ could be measured accurately (with a SNR > 44). Also, F_s_ (Ts) did not exceed the fluorescence corresponding to 0.38% fluorescein. Hence, F_s_ (Ts) measurements are also not confounded by concentration quenching.

**Fig 8:**
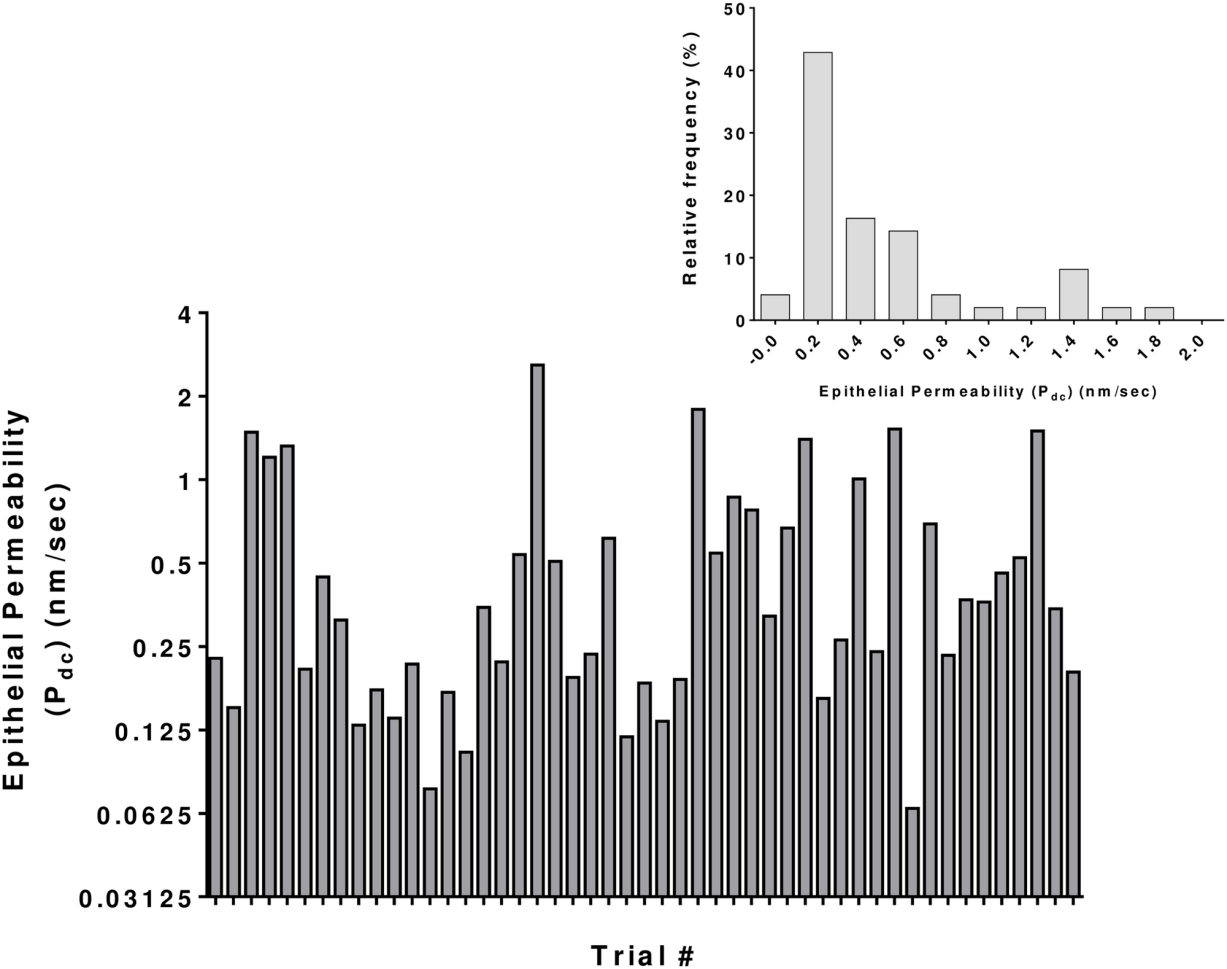
Calculated permeability of the corneal epithelium in healthy subjects. The values of P_dc_ (as calculated by Eq 22) ranged from 0.07 nm/sec to 2.59 nm/sec. The mean and SD are 0.54 and 0.54 nm/sec, respectively (n = 49 eyes and 29 subjects; median = 0.32 nm/sec).

Finally, we calculated P_dc_ for each eye by Eq 22 using estimated k_d_ and F^0^_dP_ values along with measured Fs (Ts) and Q (Raw data of all subjects is provided in S1 File). The calculated P_dc_ was 0.54 ± 0.54 nm/sec and its distribution is given by the histogram shown in Fig 8. The inset shows the histogram of P_dc_ for 49 eyes of 29 subjects. The median of P_dc_ was 0.32 nm/sec (0.07 nm/sec to 2.59 nm/sec). These calculations have been further examined by Monte Carlo simulation (i.e., MCS) [66–68] to ‘expand’ the sample size of our study and investigate the impact of the model and parameter uncertainties on the estimated P_dc_. The details are given in the S1 Appendix.

## Discussion

As a hydrophilic dye [39, 41, 72], fluorescein penetrates the corneal epithelium mainly via the paracellular pathways. Hence, changes in P_dc_ indicate either apoptosis or disruption of tight junctions. Thus, an accurate measurement of P_dc_ is of paramount importance in characterizing subtle changes in the health of the ocular surface. In addition, fluorescein is frequently employed as a fluorescent drug analog to probe the kinetics of topical ocular drug delivery [41, 71, 73, 74]. Based on these rationale, we developed a novel spot fluorometer and established a multi-drop method to reassess P_dc_ in a clinical setting.

Unlike the commonly employed commercial ocular fluorometer (i.e., Fluorotron Master^™^, Ocumetrics Inc. Palo Alto, CA), our spot fluorometer does not perform axial scans across the depth of the anterior segment. Instead, it enables measurements from any spot on the ocular surface or within the eye. As a starting point, in order to assess both the new protocol and the new spot fluorometer, we focused on measurements of P_dc_ in a cohort of healthy subjects. We have found the value of P_dc_ to be 11-fold higher than the values reported using the single-drop method [29, 37]. Although our data does not provide reasons for the observed discrepancy between estimated values by the two methods, we note that the current estimates based on the multi-drop method is closer to values reported for drugs with an octanol-water partition coefficient and molecular weight equivalent to that of fluorescein [57] (Tables 1 and 2).

### Measurements in the multi-drop method are devoid of concentration quenching

While designing the multi-drop protocol, attention was paid to two general problems of ocular fluorometry: (a) loss of fluorescence sensitivity with an increase in axial resolution of the instrument and (b) concentration quenching [29, 37]. With the excitation and emission slit widths at ~140 μm and the angle between excitation and emission arms at 45°, we found the axial resolution of our new spot fluorometer to be ~ 280 μm (Fig 2). Concentration quenching, which is dependent on the concentration of fluorescein [61], began to manifest at concentrations exceeding 0.38% for the combination of instrument settings that we employed in our measurements (Fig 2B). In experiments with Fluorotron Master^™^, others have indicated that fluorescein > 0.35% results in concentration quenching [29, 36]. Therefore, with fluorescein restricted to 0.35%, our measurements of tear fluorescence after the probe drop are devoid of nonlinearity. In other words, the estimated k_d_ and F^0^_dP_ are not confounded by concentration quenching. In contrast to the probe drop, the loading drops contained 2% fluorescein (Fig 1), which can readily cause concentration quenching (inset in Fig 2A). Therefore, we did not measure fluorescein clearance after administering the loading drops (Fig 1). In other words, k_d_ and F^0^_dL_ (shown as F^0^_dL1_ and F^0^_dL2_ in Fig 1) were not measured based on the clearance of fluorescein following the loading drops.F^0^_dL_, in particular, was obtained by scaling F^0^_dP_ (Fig 1). k_d_ is assumed to be the same between the probe and the loading drops, as confirmed in Fig 5.

Our goal for the use of the loading drops, therefore, was to secure an enhanced accumulation of fluorescein in the stroma so that stromal fluorescein levels could be measured with high SNR. As expected, the two loading drops at 2% and 6 μL led to sufficiently high F_s_, and its measurements could be performed at a high SNR of 44. Alternatively, we note that the observed standard error in the measurement of F_s_ was < 3%. Overall, the measurements of stromal fluorescence are arguably more accurate in the multi-drop protocol. Hence, the multi-drop method has the potential to yield a more precise measurement of P_dc_. Moreover, Eq 15 for the calculation of P_dc_ can easily be extended to any number of loading drops. Specifically, we can rewrite Eq 15 for “n” number of loading drops as follows:

$$P_{dc}=\frac{Q\;\beta F_{s}(T_{n+1})}{n\;\alpha [{AUC}_{dL}]}$$ (23)

where F_s_ (T_n+1_) is the average stromal fluorescence measured ~ 15 min after administration of the n^th^ drop. Increasing the number of loading drops, however, results in an increase in the overall time taken to perform P_dc_ measurements. Compared to the single-drop method, the time taken for two loading drops involves an additional 20–30 min to the protocol (Fig 1). Apart from this drawback, no other methodological problems are apparent in the new multi-drop protocol. None of the subjects showed any adverse reactions, including epithelial staining, in response to two loading drops. In hindsight, it appears that the measurement period for the probe-drop and time between the loading drops can be further compressed to reduce the overall duration of the multi-drop method. Further reduction in the duration of the protocol is also appropriate to limit the loss of fluorescein into the anterior chamber, which is negligibly small in our calculations.

### Corneal epithelial permeability is higher than previously reported

In contrast to several earlier reports based on the single-drop method and the bath technique [28, 30, 32, 35–37, 75–78], we have found mean P_dc_ to be at least 11-fold higher (Table 1). The average age of the subjects for the single-drop studies by McNamara et al. [37] was ~ 30 years, which is comparable to 28 ± 8.1 years in the current study. Thus, with age difference between the subjects in the two studies kept to a minimum, the difference in the reported values of P_dc_ is noteworthy. Our estimate of P_dc_, however, is closer to the permeability of many drugs across the corneal epithelium, especially those with molecular weights and octanol-water partition coefficients close to that of fluorescein [57–59] (Table 2). Moreover, permeability estimates based on computed partition coefficients [79] are also closer to the range of P_dc_ reported in this study. Although the discrepancy is significant, we have not been able to ascertain the cause with certainty. Additional studies in the future will unravel the definitive cause. However, the following points can be made regarding the two methods. The single-drop method results in low levels of stromal accumulation of fluorescein and hence is likely to show high variability in the measurements. In the proposed multi-drop method, the stromal accumulation is enhanced significantly relative to the single-drop method. Since the spot fluorometer is of high axial resolution (i.e., smaller focal volume), the stromal fluorescence is less likely to be confounded by levels of fluorescein in the aqueous humor. Overall, the high axial resolution in the current study and the potential for higher SNR in the stromal measurements tend to suggest that the data obtained in this study are accurate. Based on these observations, we believe that the reported value of P_dc_ could be impacted by a combination of factors including the protocol and characteristics of the instrument.

### Variability in the measured corneal epithelial permeability

Despite improvements in reducing the measurement errors, the variability in the measured P_dc_ is high, with a standard error of 0.078 (n = 49 eyes of 29 subjects). This could be mainly due to a high variability in the dynamics of fluorescein clearance (Figs 4, 6 and 7), which is affected by tear secretion, blink rate, lacrimal drainage, tear evaporation rate, and tear volume. In this context, we note that the bath technique maintains a steady concentration gradient for transport across the epithelium, and hence can avoid problems due to variability in fluorescein clearance. However, the bath technique is not suitable, as washing off fluorescein from the ocular surface and its adnexa is cumbersome and can be incomplete, leading to errors in the measurement of F_s_ (Ts).

Our initial attempts to measure P_dc_ by the single-drop method were unsuccessful because accurate measurements of F_s_ (Ts) could not be made with our spot fluorometer. As noted earlier, the relatively higher axial resolution of our instrument may have led to a reduction in sensitivity. However, 0.35% probe drop led to sufficiently high levels of the dye in the tears for an accurate assessment of k_d_ and F^0^_dP_ (Figs 4–6). Moreover, since the lock-in amplifier rejects external light, we also note that the tear fluorescence measurements were not confounded by fluctuations in the ambient light. In addition, the passage of any reflected excitation light to the photomultiplier is negligible because of the confocal optics of the spot fluorometer. This is evident in the autofluorescence values in our measurements. Thus, we conclude that the physiological variations in tear dynamics contribute to the observed variability in k_d_ and F^0^_dP_. Instrumental and observer errors are relatively small. Furthermore, to establish the robustness of our experimental P_dc_ estimate, we undertook MCS (S1 Appendix). After 10,000 iterations, as shown in Fig A1, MCS produced an estimate of P_dc_ close to that of the experimental value. If our assumptions in the derivation of Eq 22 were to be incorrect or if our experimental measurements erroneous, the MCS estimate of P_dc_ would have been significantly different from the experimental P_dc_.

### Practical implications

In addition to the clinical applications of P_dc_ as a marker of corneal epithelial health, its accurate measurement is critical for modeling drug transport across the cornea. As a hydrophilic tracer, fluorescein is commonly employed as a drug analog in evaluating drug delivery vehicles including gels and polymeric implants [71, 74]. In these situations, the necessary pharmacokinetic models could use an accurate estimate of P_dc_.

## Conclusions

In summary, we have developed a new protocol for measuring the permeability of fluorescein across the corneal epithelium using our custom-made spot fluorometer. The new fluorometer has an axial resolution of 280 μm, which is better than the resolution noted with other fluorometers [7, 12]. The measurement protocol, especially suitable for experiments with human subjects, is simple and involves a probe drop followed by two loading drops administered sequentially. In a cohort of 29 subjects (49 eyes), we have found P_dc_ to be 0.54 nm/sec. This value is 11-fold higher than the previous reports for fluorescein. However, it is close to the permeability of a wide variety of drugs with similar partition coefficients and molecular weights [58].

## Supporting information

S1 Appendix Monte Carlo simulations. (DOCX)

S1 Fig Summary of the Monte Carlo simulations. Panels A-E show distribution profiles of various parameters that produced a P_dc_ histogram similar to those of the measured values shown in the inset of Fig 8. Initially, we assumed parameters to follow either normal or Weibull distribution. Specifically, the parameters k_d,_ F^0^_dP,_ and F_s_ (Ts) were assumed to follow Weibull distribution in order to obtain a positively skewed distribution for P_dc_ similar to that observed in our experimental findings (inset of Fig 8).
(TIF)

S1 File Raw data of experiments with human subjects for epithelial permeability. Identification of the subjects has been masked with numbers. The data is provided as follows: Column A: Subject ID (masked), Column B: Sex (M: Male; F: Female), Column C: Age—rounded off (in years), Column D: 1 refers to Right Eye and 2 refers to Left Eye, Column E: CCT (um)—Central corneal thickness in micrometers as measured using OCT (Casia SS 1000), Column F: Q nm—Stromal thickness, expressed in nm, Column G: kd (per sec)—Slope; obtained by fitting decay curve of the probe drop, Column H: fdp0 –Same as F^0^_dP,_ obtained by fitting decay curve of the probe drop, Column I: Fds–Stromal fluorescence after 2 loading drops, Column J: Fds–Correction application for changes in slit width and PMT gain, Column K: ratio–Correction factor for drop size and concentration of fluorescein in the loading drop, Column L: Pdc—In nm/sec, calculated as per Eq 22 in the main text but without instrument correction factor (i.e., ICF = 47), and Column M: Pdc: In nm/sec, calculated as per Eq 22 in the main text.
(XLSX)
