# Supplementary material for: Corneal epithelial permeability to fluorescein in humans by a multi-drop method
Source: PLoS One. 2018 Jun 19;13(6):e0198831. doi: 10.1371/journal.pone.0198831 (PMC6007839; doi:10.1371/journal.pone.0198831)
Supplement: S1 Appendix — (DOCX) [file pone.0198831.s001.docx]

$\left( P_{dc} \right)$ $P_{dc}$ $Q$ $AUC$ $F_{s}$ $F_{s}$ $P_{dc}$ $P_{dc}$ $P_{dc}$ $P_{dc}$ $P_{dc}$ $P_{dc}$ $P_{dc}$ $P_{dc}$ $P_{dc}$ $P_{dc}$ $P_{dc}$ $t=0$ $T_{s}$ ${AUC}_{dL1}$ $F_{dP}^{0}$ $. k_{d}$ $F_{s}$ $P_{dc}$ $P_{dc} in Fig 8$ $P_{dc}$ $\mu$ $F_{d}^{A}$ $C_{F}$ $F_{d}^{B}$ $F_{d}^{A}.$ $F_{s}^{C}$ $C_{F}$ $2\delta.$ $\left( V_{i}^{0} \right)$ $C_{dP}(t)$ $C_{dP}\left( t \right)= C_{dP}^{0}e^{-k_{d}t}$ $C_{dP}^{0}$ $t=0$ $C_{dP}^{0}$ $k_{d}$ $C_{dP}\left( t \right)= \alpha F_{dP}$ $\alpha$ $C_{dP}^{0}= \alpha F_{dP}^{0}$ $\alpha$ $F_{dP}\left( t \right)= F_{dP}^{0}e^{-k_{d}t}$ $\left[ {AUC}_{P} \right]=F_{dP}^{0}\int_{0}^{T} e^{-k_{d}t} dt= \frac{F_{dP}^{0}}{k_{d}}\left( 1-e^{-k_{d}T} \right)$ $\left[ {AUC}_{P} \right]≝\int_{0}^{T} F_{dP} dt$ $5\times t_{1/2}^{d}$ $\left[ {AUC}_{P} \right]= \frac{F_{dP}^{0}}{k_{d}}$ $k_{d}$ $F_{dP}^{0}$ $k_{d}$ $t_{1/2}^{d}$ $k_{d}$ $k_{d}$ $P_{dc}$ $P_{dc}$ $\frac{dm_{s}}{dt}≝P_{dc} A \left( C_{dL1}-C_{s} \right), where m_{s}=Q AC_{s}$ $m_{s}$ $A$ $Q$ $\left( Q\times A \right)$ $C_{s}$ $C_{d}$ $C_{dL1}$ $m_{s}$ $C_{s}$ $C_{dL1}$ $Q\frac{dC_{s}}{dt}=P_{dc}C_{dL1}$ $F_{s}$ $C_{s}\left( t \right)= \beta F_{s}$ $\beta$ $\beta Q\frac{dF_{s}}{dt}=P_{dc}{\alpha F}_{dL1}$ $\alpha$ $\beta$ $T_{1}$ $T_{2}$ $\beta Q \left[ F_{s}\left( T_{2} \right)- F_{s}\left( T_{1} \right) \right]= P_{dc} \alpha\left[ {AUC}_{dL1} \right]$ $\left[ {AUC}_{dL1} \right]≝\int_{T_{1}}^{T_{2}} F_{dL1} dt$ $T_{2}$ $\beta Q \left[ F_{s}\left( T_{3} \right)- F_{s}\left( T_{2} \right) \right]= P_{dc} \alpha\left[ {AUC}_{dL2} \right]$ $\left[ {AUC}_{dL2} \right]≝\int_{T_{2}}^{T_{3}} F_{dL2} dt$ $\beta Q\left[ F_{s}\left( T_{2} \right)- F_{s}\left( T_{1} \right)+ F_{s}\left( T_{3} \right)- F_{s}\left( T_{2} \right) \right]$ $T_{1}$ $T_{2}$

$$\left[ {AUC}_{dL2} \right]= \left[ {AUC}_{dL1} \right] = \left[ {AUC}_{dL} \right]$$

$\beta Q\left[ - F_{s}\left( T_{1} \right)+ F_{s}\left( T_{3} \right) \right]$ $T_{1}$ $F_{s}\left( T_{1} \right)$ $P_{dc}= \frac{Q \beta F_{s}\left( T_{3} \right)}{2 \alpha\left[ {AUC}_{dL} \right]}$ $F_{s}\left( T_{3} \right)$ $T_{s}$ $\alpha$ $F_{d}$ $C_{d}$ $C_{d}=\alpha\times F_{d}$ $\beta$ $F_{s}$ $C_{s}$ $C_{s}=\beta\times F_{s}$ $\alpha$ $\beta$ $T_{3}$ $\alpha,$ $\left[ {AUC}_{dL} \right]$ $P_{dc}$ $\left[ {AUC}_{dL} \right]$ $V_{i}$ $V_{d}$ $M_{P}$ $C_{dP}^{0}= \frac{M_{P}}{V_{i} + V_{d}}$ $M_{L}$ $C_{dL}^{0}= \frac{M_{L}}{{Vi+V}_{d}}$ $C_{dP}^{0}= {\alpha F}_{dP}^{0}$ $F_{dP}^{0}=\frac{M_{P}}{\alpha\left( 2+V_{d} \right)}$ $F_{dP}^{0}$ $F_{dL}^{0}=\frac{M_{L}}{\alpha\left( 6+V_{d} \right)}$ $\frac{F_{dL}^{0}}{F_{dP}^{0}}= \frac{M_{L}}{M_{P}}\frac{{(2+V}_{d})}{{(6+V}_{d})}$ $\left[ {AUC}_{dL} \right]$

$$\left[ {AUC}_{dL} \right]= \frac{F_{dL}^{0}}{k_{d}}$$

$F_{dL}^{0}$ $\left[ {AUC}_{dL} \right]= F_{dP}^{0}\frac{1}{k_{d}}\frac{M_{L}}{M_{P}}\frac{{(2+V}_{d})}{{(6+V}_{d})}$ $\left[ {AUC}_{dL} \right]$ $P_{dc}$ $F_{dP}^{0}$ $P_{dc}$ $P_{dc}= \frac{k_{d}Q\beta F_{s}\left( T_{3} \right)}{2 \alpha F_{dP}^{0}}\frac{M_{P}}{M_{L}}\frac{{(6+V}_{d})}{{(2+V}_{d})}$ $P_{dc}$ $\alpha$ $M_{P}$ $M_{L}$ $k_{d}$ $F_{dP}^{0}$ $P_{dc}$ $F_{dP}^{0}$ $k_{d}$ $F_{dP}^{0})$ $({F^{0}}_{dP}$ ${F^{0}}_{dP}$ $k_{d}$ $k_{d}$ $P_{dc}$ $F_{s} (T1))$ $F_{s} \left( T3 \right)$ $F_{s}$ $F_{s} \left( Ts \right)$ $F_{s} \left( Ts \right)$ $P_{dc}$ $k_{d}$ $F_{dP}^{0}$ $F_{s} \left( Ts \right)$ $Q$ $P_{dc}$ $P_{dc}$ $P_{dc}$ $P_{dc}$ $P_{dc}$ $P_{dc}$ $P_{dc}$ $P_{dc}$ $P_{dc}$ $k_{d}$ $F_{dP}^{0}$ $k_{d}$ $F_{dL}^{0}$ $F_{dL1}^{0}$ $F_{dL2}^{0}$ $F_{dP}^{0}$ $k_{d}$ $F_{s}$ $F_{s}$ $P_{dc}$ $P_{dc}$ $P_{dc}= \frac{Q \beta F_{s}\left( T_{n+1} \right)}{n \alpha\left[ {AUC}_{dL} \right]}$ $F_{s}\left( T_{n+1} \right)$ $P_{dc}$ $P_{dc}$ $P_{dc}$ $P_{dc}$ $P_{dc}$ $P_{dc}$ $P_{dc}$ $F_{s} \left( Ts \right)$ $k_{d}$ $F_{dP}^{0}$ $k_{d}$ $P_{dc}$ $P_{dc}$ $P_{dc}$ $P_{dc}$ $P_{dc}$ $P_{dc}$**S1 Appendix:**

**Monte Carlo Simulation**

Our experimental $P_{dc}$P_dc_ estimate, which is computed using Eqn. 22, is based on a set of measurements with each of the 49 eyes. Eqn. 22, which forms the mathematical model for the multi-drop protocol, is derived based on several assumptions. Therefore, the associated modeling uncertainties contribute to the errors or variability in the computed P_dc_. In addition, the computed $P_{dc}$P_dc_ is confounded by errors in measurements (Q, F_s_ (Ts)$Q, F_{s} \left( Ts \right)$), which are necessary to calculate the parameters (k_d_$k_{d}, F_{dP}^{0}$ F^0^_dP_) of Eq. 22. Moreover, we have assumed $V_{d}$ V_d_ as a constant, but it may vary among subjects. In this context, we have performed virtual experiments (i.e., Monte Carlo Simulations, MCS) [66-68] to ‘expand’ the sample size of our study and demonstrate the impact of model and parameter uncertainties on the $P_{dc}$P_dc_ estimate.

For each iteration of the MCS, we randomly sampled k_d,_ F^0^_dP_ $k_{d}, F_{dP}^{0}$ and $F_{s} \left( Ts \right)$ F_s_ (Ts) from three different Weibull distributions, while $Q$Q and $V_{d}$ V_d_ were drawn from two different Normal distributions. Table A1 specifies the distributions describing each of the parameters. Note that for parameters with Normal distribution, the mean and standard deviation were close to the corresponding experimental values. For parameters assigned to a Weibull distribution, the mean was set close to the corresponding experimental value. We carried out 20,000 iterations and after each iteration of sampling the parameters/measurements, we calculated $P_{dc}$P_dc_ using Eq. 22. Panels A-E of S1 Fig show the histograms of various parameters randomly chosen for the 20, 000 iterations. Finally, the calculated P_dc_$P_{dc}$from all iterations were analyzed for mean and standard deviation. As shown in S1 Fig (Panel F), the mean of the P_dc_$P_{dc}$estimate by the MCS is reasonably close to the experimental$P_{dc}$ P_dc_. Moreover, less than ~10% of the subjects in the MCS show P_dc_$P_{dc}$below 0.1 nm/sec. These findings suggest that Eq. 22 adequately represents the permeability of fluorescein into the stroma during the multi-drop protocol, and indicate the robustness of the experimental $P_{dc}$P_dc_ estimate.

**Table A1. Parameters for the Monte Carlo Simulation.** The mean values for all parameters are close to the experimental values. The standard deviation and the actual probability distribution functions (PDF; Normal and Weibull) were varied to fit the experimental distribution of P_dc_$P_{dc}$ shown in the inset of Fig 8. PDFs were computed using the library (as SubVIs) provided with LabVIEW (2014).

| Parameter | Probability Distribution | Mean | PDF Parameters | Units |
| --- | --- | --- | --- | --- |
| $k_{d}$ k_d_ | Weibull distribution | 0.009 | α = 3 β = 25 | sec^-1^ |
| $F_{dP}^{0}$ F^0^_dP_ | Weibull distribution | 197 | α = 3 β = 25 | mV |
| $Q$Q | Normal distribution | 476 | σ = 28 | µm |
| $V_{d}$ V_d_ | Normal distribution | 8 | σ = 3 | µL |
| $F_{s} \left( Ts \right)$ F_s_ (Ts) | Weibull distribution | 8.2 | α = 3 β = 25 | mV |
| Experimental $P_{dc}$ P_dc_ |  | 0.54  Range ~0.07 – 2.59  Median ~ 0.32 | σ = 0.54 | nm/sec |
| Estimated $P_{dc}$ P_dc_ | Monte Carlo Simulation (20000 iterations) | 0.56  Median ~ 0.47 | σ = 0.43 | nm/sec |

**S1 Fig : Summary of the Monte Carlo simulations:** Panels A-E show distribution profiles of various parameters that produced a $P_{dc}$P_dc_ histogram similar to those of the measured values shown in the inset of Fig 8. Initially, we assumed parameters to follow either normal or Weibull distribution. Specifically, the parameters k_d,_ F^0^_dP,_ and F_s_ (Ts)$k_{d}, F_{dP}^{0}, and F_{s}(Ts)$ were assumed to follow Weibull distribution in order to obtain a positively skewed distribution for $P_{dc}$ P_dc_ similar to that observed in our experimental findings (inset of Fig 8).
