# Supplementary figures and images for: Corneal epithelial permeability to fluorescein in humans by a multi-drop method
Source: PLoS One. 2018 Jun 19;13(6):e0198831. doi: 10.1371/journal.pone.0198831 (PMC6007839; doi:10.1371/journal.pone.0198831)

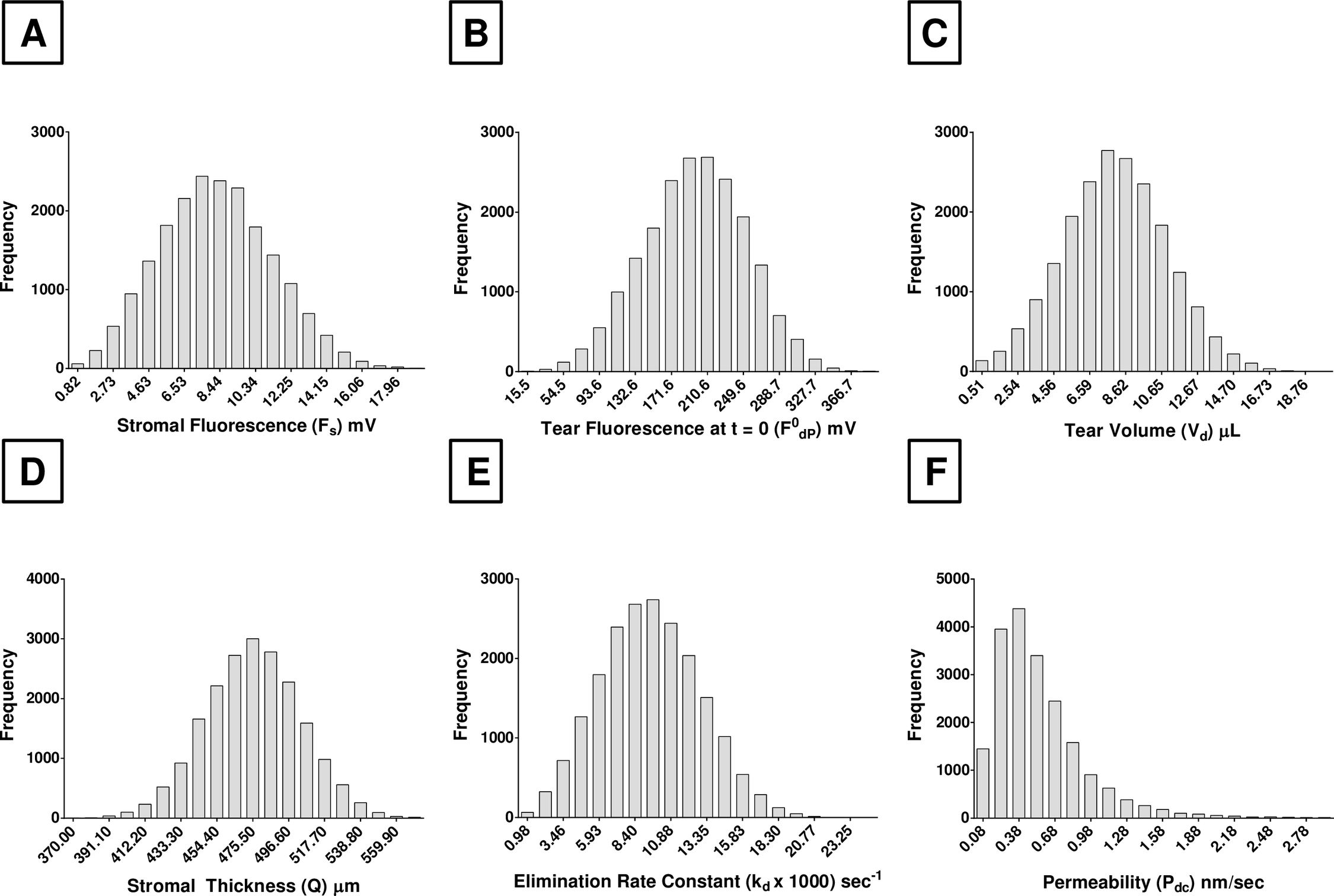

Supplement: S1 Fig — Panels A-E show distribution profiles of various parameters that produced a Pdc histogram similar to those of the measured values shown in the inset of Fig 8. Initially, we assumed parameters to follow either normal or Weibull distribution. Specifically, the parameters kd, F0dP, and Fs (Ts) were assumed to follow Weibull distribution in order to obtain a positively skewed distribution for Pdc similar to that observed in our experimental findings (inset of Fig 8). (TIF) [file pone.0198831.s002.tif]
